# Synthesis, Tautomeric Structure and Antimicrobial Activity of 3-Arylhydrazono-4-phenyl-[1,2,4]-triazepino[2,3-a]quinazoline-2,7(1*H*)-diones

**DOI:** 10.3390/molecules17078483

**Published:** 2012-07-13

**Authors:** Thoraya Abdel Reheem Farghaly, Mastoura Mohamed Edrees, Mosselhi Abdelnabi Mosselhi

**Affiliations:** 1 Department of Chemistry, Faculty of Science, University of Cairo, Giza 12311, Egypt; Email: Thoraya-f@hotmail.com; 2 Department of Organic Chemistry, National Organization for Drug Control and Research (NODCAR), Giza 12311, Egypt; Email:mmohamededrees@yahoo.com; 3 Department of Chemistry, Faculty of Science, Taif University, Taif-888, Saudi Arabia

**Keywords:** azo-hydrazone, tautomerism, [1,2,4]triazepino[2,3-*a*]quinazolindione, anti-microbial activity

## Abstract

A simple strategy for the synthesis of the hitherto unreported 3-arylazo-4-phenyl- [1,2,4]triazepino[2,3-*a*]quinazoline-2,7(1*H*)-diones is described. Spectral data indicated that the studied compounds exist predominantly in the hydrazone tautomeric form. The antimicrobial activity of the newly synthesized compounds was also evaluated. The results indicated that some of these compounds have moderate activity towards bacteria.

## 1. Introduction

4(*3H*)-Quinazolinone has to be the most used heterocycle in medicinal chemistry [1,2,3] and it is considered as a key building block for natural alkaloids [4]. A wide spectrum of biological effects is reported for compounds containing the quinazoline ring system. For example, many quinazoline derivatives show antifungal [5], antitoxoplasmosis [6], anti-HIV-I agent [7], antitumour [8,9,10], anti-allergic and antihypertensive properties [11]. They also have been used to prevent ocular inflammatory reactions [12]. 2-Thioxo-4(1*H*)-quinazolinones are important derivative of quinazolinones found to possess a variety of bioactivities and are good precursors for the synthesis of fused quinazolines [13,14,15]. 2,3-Diaminoquinazolinone is reported to be the key for synthesis of derivatives of thiazolo[4,3-*b*]benzothiazole, thiazolo[3',4':2,3], [1,2,4]-triazolo[5,1-*b*]-, {triazino[3,2-*b*]- and triazepino[3,2-b]}-quinazolines [16]. 2-Thioxo-1*H*-4-quinazolinones are also versatile intermediates for fused heterocyclo quinazolinones like 2-phenyl-5*H*-[1,3,4]thiadiazolo[2,3-b]quinazolin-5-one, 3-(4-bromophenyl)-2*H*,6*H*-[1,3,4]thiadiazino-[2,3-b]quinazolin-6-one, 4-amino-2-phenyl-3a,4-dihydro-2*H*-thiazolo[3,2*a*]quinazoline -1,5-dione and 2-phenyl-[1,3,4]thiadiazino[2,3-*b*]quinazoline-3,6(2*H*,4*H*)-dione [17].

It has been reported recently that the azo-hydrazone tautomerism of diazonium coupling products of acyclic and heterocyclic active methylene compounds in both ground and excited states is useful in the field of material sciences [18,19,20,21,22,23,24].

**Scheme 1:**
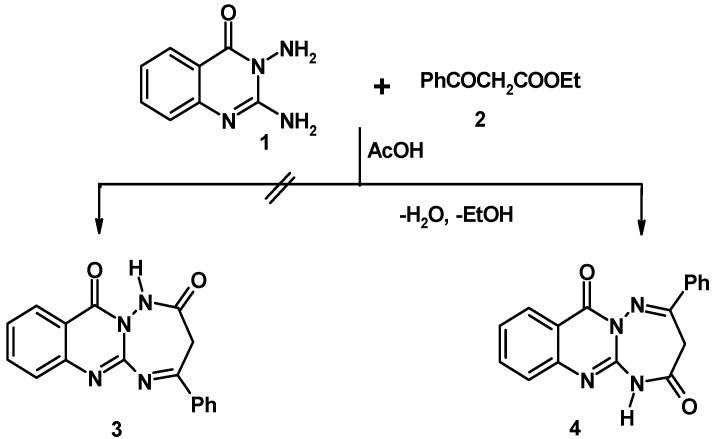
Synthesis of 4-phenyl-1,3-dihydro[1,2,4]triazepino[2,3-a]quinozalin-2,7-dione (**4**).

**Scheme 2:**
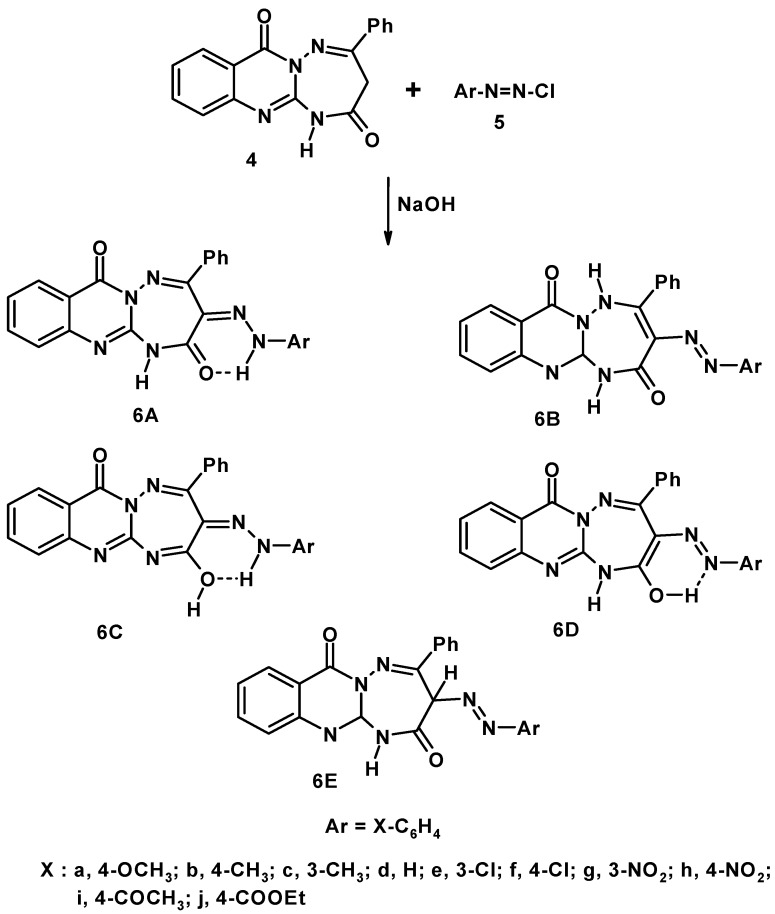
Synthesis of 3-arylhydrazino-4-phenyl-[1,2,4]triazepino[2,3-*a*]quinazolin-2,7(1*H*)-dione (**6a–j**).

**Figure 1:**
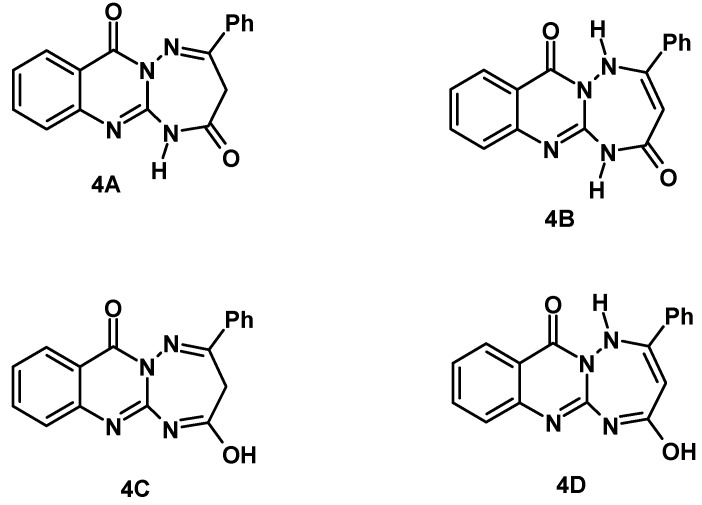
Four tautomeric structures **A–D** of **4**.

Due to the forgoing importance of quinazoline derivatives and as a part of our program of studies on the chemistry of bioactive molecules and azo-hydrazone tautomerism [18,19,20,21,22,23,24], it was thought interesting to study the synthesis of 4-phenyl-1,3-dihydro[1,2,4]triazepino[2,3-a]quinozalin-2,7-dione (**4**) (Scheme 1) and its reaction with diazonium salts **5** in an attempt to synthesize the respective title compounds, 3-arylhydrazino-4-phenyl-[1,2,4]triazepino[2,3-*a*]quinazolin-2,7(1*H*)-diones, **6** (Scheme 2) and to study their tautomeric structures. This is because such products can have one or more of the four tautomeric structures **A–D** shown in Figure 1. In addition, the products **6** obtained were tested for their biological activity which is also reported.

## 2. Results and Discussion

The starting 4-phenyl-1,3-dihydro-[1,2,4]triazepino[2,3-*a*]quinazoline-2,7-dione **4** was prepared by condensation of 2,3-diamino-quinazolin-4-one **1** [25] with ethyl benzoylacetate **2** in acetic acid under reflux. The structure of **4** was indicated by its elemental and spectral (IR, ^1^H-NMR and MS) analyses (see the experimental section). As shown in Scheme 1, the reaction of **1** with **2** could in principle yield **4** and/or its isomer, namely, 2-phenyl-3,5-dihydro[1,2,4] triazepino[2,3-*a*]quinazoline-4,7-dione (**3)**, but the latter isomeric structure was discarded. because condensation reactions of ethyl benzoylacetate with C,N-1,2-diaminoheterocycles thus far studied were reported to be regioselective and lead to products that result from nucleophilic attack of the N-NH_2_ and the C-NH_2_ groups at the keto and ester carbonyl groups of the β-keto ester, respectively to give the corresponding fused 4-oxo-1,2,4-triazepine derivatives [26,27,28]. This regioselectivity was substantiated by X-ray crystallographic analysis in some cases [28] and by spectral data [26,27,28].

The structure **4** can exist in one of the possible tautomeric forms **4A–D** as shown in Figure 1. The isomeric form **4A** is the predominant one based on the spectral data (MS, ^1^H-NMR and IR). For example, the ^1^H-NMR spectrum revealed three characteristic signals near δ 4.52, 7.38–8.22 and 13.15 which are assignable to CH_2_, C_6_H_5_ and the ring NH protons, respectively. Also, the mass spectrum of compound **4** showed the molecular ion peak at *m/z* 304, which is consistent with its structural formula.

Reaction of **4** with diazotized anilines in basic medium afforded the respective arylazo derivatives **6** (Scheme 2). The mass spectra of the latter products revealed the molecular ion peaks at the expected *m/z* values and their elemental analyses data were consistent with their assigned structures. The infrared spectral data of compounds **6** (see Experimental) seem to be consistent more with the hydrazone tautomeric form **6A** rather than the other tautomeric forms **6B–E** (Scheme 2). For example, all compounds exhibit two carbonyl bands in the regions 1715–1681 and 1698–1643 cm^−1^ corresponding to the stretching vibrations of the pyrimidinone and the 1,2,4-triazepinone carbonyl groups, respectively. The observed wave number of the latter CO stretching band in compounds **6** seems to result from the possible strong hydrogen bond with the hydrazone NH and conjugation with the C=N double bond as required by the hydrazone form **6A** (Scheme 2) [29].

To elucidate the actual tautomeric form of the studied compounds **6**, we investigated their electronic absorption spectra. The data are summarized in Table 1. As shown, each of compounds **6** in dioxane exhibits three characteristic absorption bands in the regions 385–346, 328–248 and 257–231 nm. Such an absorption pattern is similar to that of typical hydrazone chromophore [23,24]. Furthermore, the spectrum of **6d**, taken as a typical example of the series studied was recorded in variety solvents with different polarities. The spectra obtained showed little, if any, shift (Table 1).

The small shifts in λ_max_ of **6d** in different solvents are due to solute-solvent interaction. In agreement with this conclusion, is the observation that the spectra of arylhydrazones derived from the reaction of quinones with *N*-alkyl-*N*-phenylhydrazine, unlike those of *o*- and *p*-hydroxyazo compounds, are largely independent of the solvent polarity [30]. This finding, while it excludes the azo tautomeric forms **6B**, **6D** and **6E**, indicates that each of compounds **6** exist in one tautomeric form, namely **6A**(Scheme 2).

**Table 1:** UV Spectra of 3-arylhydrazono-4-phenyl-1*H*-[1,2,4]triazepino[2,3-*a*]quinazolin-2,7-diones **6a–j** in dioxane.

Compd. No.	λ_max_ (log ε)
**6a**	385 (3.72), 321 (4.22), 257 (4.63)
**6b**	381 (4.52), 260 (4.57), 231 (4.78)
**6c**	375 (4.02), 282 (4.82), 247 (4.81)
**6d ** **^a)^**	370 (4.45), 248 (4.63)
**6e**	372 (4.25), 286 (4.26), 231 (4.55)
**6f**	372 (4.10), 328 (4.39), 233 (5.13)
**6g**	346 (3.62), 267 (4.78), 232 (5.01)
**6h**	383 (4.62), 260 (4.54), 234 (4.80)
**6i**	372 (3.94), 260 (3.68), 235 (4.01)
6j	381 (4.52), 260 (4.57), 231 (4.78)

^a)^ Solvent λ_max_ (logε): acetic acid 370 (4.36), 248 (4.00); acetonitrile 370 (3.80), 246 (4.11); DMF 367 (4.01), 247 (4.14); ethanol 371 (3.60), 249 (3.70).

### 2.1. Anti-Microbial Activity

*In vitro* anti-microbial screening of compounds **4** and **6a–j** prepared in this study was carried out using two bacteria species, including *a* Gram negative bacterium, *Escherichia coli (RCMB 000103) (EC)* and a Gram positive bacterium*, Staphylococcus aureus (RCMB 000106) (SA)* and twofungal strains, namely *Aspergillus fumigatus (RCMB 002003)* (AF) and *Candida albicans (RCMB 005002) (CA)* using the agar diffusion well method (see Experimental). The microorganisms were tested against the activity of solutions of concentrations of 1.0 mg/mL of each compound in dimethylsulfoxide (DMSO) and using an inhibition zone diameter in cm (IZD) as criterion for the antimicrobial activity. The fungicide amphotericin B and the bactericide tetracycline were used as references to evaluate the potency of the tested compounds under the same conditions. The results, depicted in Table 2, showed that compounds **6a**, **e**, **h–j** displayed moderate activities against *E. coli* and *S. aureus*, while, none of the tested compounds have any activity against *A.* *fumigatus* and *C. albicans*. These biological activities of compounds **6a–j** are far less than the studied reference drugs (tetracycline and amphotericin B). 

**Table 2:** Antimicrobial Activity of the newly synthesized compounds.

	*Microorganism*/IZD (mm/mg sample) *			
Cpd. no.	*EC(G^−^)*	*SA (G^+^)*	*AF*	*CA*
**Control (DMSO)**	0.0	0.0	0.0	0.0
**4**	00	9	00	0.0
**6a**	12	11	0.0	0.0
**6b**	00	00	0.0	0.0
**6c**	00	00	00	00
**6d**	00	00	00	00
**6e**	13	12	00	00
**6f**	00	00	00	00
**6g**	9	00	00	00
**6h**	11	12	00	00
**6i**	11	11	00	00
**6j**	12	12	00	00
**Te ** **	31	29	--	--
**Am ** **	--	--	17	20

* IZD, inhibition zone diameter, ** Te = Tetracycline used as standard antibacterial agent and ** Am = Amphotericin B used as standard antifungal agent.

## 3. Experimental Section

### 3.1. General

Melting points were determined on a Gallenkamp apparatus and are uncorrected. IR spectra were recorded in potassium bromide pellets using Perkin Elmer FTIR 1650 and Pye-Unicam SP300 infrared spectrophotometers. ^1^H-NMR and ^13^C-NMR spectra were recorded in deuterated dimethyl sulfoxide (DMSO-d_6_) using a Varian Gemini 300 NMR spectrometer. Mass spectra were recorded on a GCMS-QP 1000 EX Shimadzu and GCMS 5988-A HP spectrometers. Electronic absorption spectra were recorded on Perkin-Elmer Lambada 40 spectrophotometer. Elemental analyses were carried out at the Microanalytical Laboratory of Cairo University, Giza, Egypt. 2,3-Diamino-quinazoline-4-one **1** was prepared as previously described [25].

*4-Phenyl-1,3-dihydro-[1,2,4]triazepino[2,3-a]quinazoline-2,7-dione* (**4**)*.* A mixture of 2,3-diamino-quinazolin-4-one (**1**, 1.76 g, 10 mmol) and ethyl benzoylacetate (**2**, 2.4 g, 3 mL, 20 mmol) in acetic acid (20 mL) was refluxed for 30 h and cooled. The solid that precipitated was collected and crystallized from ethanol to give compound **4** as yellow crystalline solid, yield (40%). mp. 282–284 °C. ^1^H-NMR (DMSO-d_6_) δ 4.56 (s, 2H, CH_2_), 7.35–8.22 (m, 9H, ArH), 13.15 (s, 1H, NH). MS *m/z* (%) 305 (M^+^ + 1, 1), 304 (M^+^, 10), 176 (13), 157 (18), 144 (32), 104 (46), 77 (77), 76 (100). Anal. Calcd for C_17_H_12_N_4_O_2_ (304.30): C, 67.10; H, 3.97; N, 18.41; Found: C, 67.24; H, 3.86; N, 18.25%.

### 3.2. General Procedure for Synthesis of 3-Arylhydrazono-4-phenyl-[1,2,4]triazepino[2,3-a]quinazoline-2,7(1H)-dione*s (**6a–j**)*.

To a stirred solution of compound **4** (1.52 g, 5 mmol) in ethanol (40 mL) was added sodium hydroxide (0.2 g, 5 mmol) and the mixture was cooled in an ice bath at 0–5 °C. To the resulting solution, while being stirred, was added dropwise over a period of 20 min, a solution of the appropriate arenediazonium chloride, prepared as usual by diazotizing the respective aniline (5 mmol) in hydrochloric acid (6 M, 3 mL) with sodium nitrite (1 M, 5 mL). The whole mixture was then left in a refrigerator overnight. The precipitated solid was collected, washed with water and finally crystallized from ethanol to give the respective hydrazones **6a–j**.

3*-(4-Methoxyphenylhydrazono)-4-phenyl-[1,2,4]triazepino[2,3-a]quinazoline-2,7(1H)-dione* (**6a**). This compound was obtained as red solid, (68%). mp. 246–248 °C. ^1^H-NMR (DMSO-d_6_) δ 3.82 (s, 3H, OCH_3_), 6.92 (d, *J* = 9 Hz, 2H, ArH), 7.07–7.910 (m, 9H, ArH), 7.92 (d, *J* = 9 Hz, 2H, ArH), 12.20 (s, 1H, NH), 13.44 (s, 1H, NH); IR (KBr) ν_max_ 3400, 3147 (2NH), 1705, 1681 (2CO) cm^−1^. MS *m/z* (%) 439 (M^+^ + 1, 2), 438 (M^+^, 7), 122 (14), 105 (100), 77 (41). Anal. Calcd for C_24_H_18_N_6_O_3_ (438.44): C, 65.75; H, 4.14; N, 19.17; Found: C, 65.64; H, 4.10; N, 19.09%.

*3-(4-Methylphenylhydrazono)-4-phenyl-[1,2,4]triazepino[2,3-a]quinazoline-2,7(1H)-dione* (**6b**). This compound was obtained as brown solid, (69%). mp. 232–236 °C. ^1^H NMR (DMSO-d_6_) δ 2.45 (s, 3H, CH_3_), 7.05 (d, *J* = 8 Hz, 2H, ArH), 7.14–7.94 (m, 9H, ArH), 8.26 (d, *J* = 8 Hz, 2H, ArH), 12.07 (s, 1H, NH), 13.84 (s, 1H, NH); ^13^C NMR(DMSO-d_6_) δ 20.30, 113.39, 114.36, 115.87, 116.63, 121.40, 122.73, 125.15, 126.52, 127.28, 127.99, 128.93, 129.91, 131.48, 132.09, 138.53, 140.31, 159.41, 163.77, 164.25. IR (KBr) ν_max_ 3471, 3406 (2NH), 1697, 1651 (2CO) cm^−1^. MS *m/z* (%) 422 (M^+^, 31), 317 (5 ), 211 (7), 105 (100), 90 (18), 77 (44). Anal. Calcd for C_24_H_18_N_6_O_2_ (422.44): C, 68.24; H, 4.29; N, 19.89; Found: C, 68.17; H, 4.31; N, 19.74%.

*3-(3-Methylphenylhydrazono)-4-phenyl-[1,2,4]triazepino[2,3-a]quinazoline-2,7(1H)-dione* (**6c**). This compound was obtained as yellow solid, (59%). mp. 200–204 °C. ^1^H-NMR (DMSO-d_6_) δ 2.24 (s, 3H, CH_3_), 6.84–8.30 (m, 13H, ArH), 11.90 (s, 1H, NH), 13.33 (s, 1H, NH); IR (KBr) ν_max_ 3433, 3100 (2NH), 1712, 1685 (2CO) cm^−1^. MS *m/z* (%) 422 (M^+^, 10), 211 (11), 130 (10), 105 (100), 90 (12), 77 (55). Anal. Calcd for C_24_H_18_N_6_O_2_ (422.44): C, 68.24; H, 4.29; N, 19.89; Found: C, 68.10; H, 4.21; N, 19.68%.

*3-Phenylazohydrazono-4-phenyl-[1,2,4]triazepino[2,3-a]quinazoline-2,7(1H)-dione* (**6d**). This compound was obtained as yellow solid, (82%). mp. 244–246 °C. ^1^H NMR (DMSO-d_6_) δ 7.03–8.30 (m, 14 H, ArH), 11.90 (s, 1H, NH), 13.50 (s, 1H, NH); ^13^C NMR (DMSO-d_6_) δ 112.31, 115.23, 117.11, 120.95, 123.06, 124.19, 125.06, 126.34, 128.34, 129.07, 131.11, 132.26, 136.44, 138.60, 141.36, 156.20, 159.92, 161.23, 162.15. IR (KBr) ν_max_ 3471, 3178 (2NH), 1700, 1654 (2CO) cm^−1^. MS *m/z* (%) 408 (M^+^, 64), 407 (71), 144 (25), 134 (15), 105 (84), 91 (100), 77 (76). Anal. Calcd for C_23_H_16_N_6_O_2_ (408.41): C, 67.64; H, 3.95; N, 20.58; Found: C, 67.50; H, 4.09; N, 20.38%.

*3-(3-Chlorophenylhydrazono)-4-phenyl-[1,2,4]triazepino[2,3-a]quinazoline-2,7(1H)-dione* (**6e**). This compound was obtained as yellow solid, (75%). mp. 180–182 °C. ^1^H-NMR (DMSO-d_6_) δ 7.34–8.19 (m, 13H, ArH), 11.32 (s, 1H, NH), 13.22 (s, 1H, NH); IR (KBr) ν_max_ 3401, 3100 (2NH), 1689, 1654 (2CO) cm^−1^. MS *m/z* (%) 444 (M^+^ + 2, 1), 443 (M^+^ + 1, 4), 442 (M^+^, 3), 211 (15), 122 (16), 111 (6), 104 (100), 90 (10), 76 (50). Anal. Calcd for C_23_H_15_ClN_6_O_2_ (442.86): C, 62.38; H, 3.41; N, 18.98; Found: C, 62.29; H, 3.34; N, 18.79%. 

*3-(4-Chlorophenylhydrazono)-4-phenyl-[1,2,4]triazepino[2,3-a]quinazoline-2,7(1H)-dione* (**6f**). This compound was obtained as yellow solid, (80%). mp. 240–242 °C. ^1^H-NMR (DMSO-d_6_) δ 7.12–7.95 (m, 9H, ArH), 8.05 (d, *J* = 8 Hz, 2H, ArH), 8.18 (d, *J* = 8 Hz, 2H, ArH), 11.82 (s, 1H, NH), 13.20 (s, 1H, NH); ^13^C NMR(DMSO-d_6_) δ 115.23, 117.17, 118.01, 122.84, 125.29, 126.20, 127.03, 128.42, 129.18, 129.56, 131.30, 132.16, 137.54, 138.19, 142.30, 154.36, 158.90, 162.21, 163.56. IR (KBr) ν_max_ 3282, 3186, (2NH), 1681, 1647 (2CO) cm^−1^. MS *m/z* (%) 444 (M^+^ + 2, 2), 442 (M^+^, 5), 304 (83), 276 (33), 130 (11), 111 (2), 105 (100), 89 (14), 76 (87). Anal. Calcd for C_23_H_15_ClN_6_O_2_ (442.86): C, 62.38; H, 3.41; N, 18.98; Found: C, 62.19; H, 3.25; N, 18.88%. 

*3-(3-Nitrophenylhydrazono)-4-phenyl-[1,2,4]triazepino[2,3-a]quinazoline-2,7(1H)-dione* (**6g**). This compound was obtained as yellowish brown solid, (64%). mp. 286–288 °C. ^1^H-NMR (DMSO-d_6_) δ 7.10–8.15 (m, 12H, ArH), 8.33 (s, 1H, ArH), 11.39 (s, 1H, NH), 13.61 (s, 1H, NH); IR (KBr) ν_max_ 3440, 3224 (2NH), 1700, 1651 (2C=O) cm^−1^. MS *m/z* (%) 453 (M^+^, 15), 144 (12), 118 (15), 105 (100), 91 (23), 76 (54). Anal. Calcd for C_23_H_15_N_7_O_4_ (453.41): C, 60.93; H, 3.33; N, 21.62; Found: C, 60.85; H, 3.21; N, 21.49%.

*3-(4-Nitrophenylhydrazono)-4-phenyl-[1,2,4]triazepino[2,3-a]quinazoline-2,7(1H)-dione* (**6h**). This compound was obtained as dark red solid, (76%). mp. 268–270 °C. ^1^H-NMR (DMSO-d_6_) δ 7.13–7.30 and 7.44–8.01 (m, 9H, ArH), 7.33 (d, *J* = 8 *Hz*, 2H, ArH), 8.27 (d, *J* = 8 *Hz*, 2H, ArH), 11.54 (s, 1H, NH), 13.52 (s, 1H, NH);^ 13^C NMR(DMSO-d_6_) δ 113.95, 120.77, 125.22, 127.30, 128.41, 128.89, 130.0, 132.29, 133.46, 134.71, 135.31, 136.61, 141.56, 143.02, 144.21, 148.78, 159.0, 162.26, 162.85. IR (KBr ν_max_ 3440, 3379 (2NH), 1698, 1658 (2C=O) cm^−1^. MS *m/z* (%) 454 (M^+^ + 1, 2), 453 (M^+^, 25), 211 (13), 145 (9), 105 (100), 76 (52). Anal. Calcd for C_23_H_15_N_7_O_4_ (453.41): C, 60.93; H, 3.33; N, 21.62; Found: C, 60.79; H, 3.25; N, 21.71%.

*3-(4-Acetylphenylhydrazono)-4-phenyl-[1,2,4]triazepino[2,3-a]quinazoline-2,7(1H)-dione* (**6i**). This compound was obtained as reddish brown solid, (71%). mp. 176–178 °C. ^1^H-NMR (DMSO-d_6_) δ 2.25 (s, 3H, COCH_3_), 7.08 (d, *J* = 8 *Hz*, 2H, ArH), 7.38–7.94 (m, 9H, ArH), 8.06 (d, *J* = 8 *Hz*, 2H, ArH), 11.96 (s, 1H, NH), 12.40 (s, 1H, NH); ^13^C-NMR (DMSO-d_6_) δ 55.29, 114.0, 114.60, 114.89, 115.32, 119.32, 125.05, 126.10, 127.91, 129.32, 129.83, 131.82, 132.11, 136.69, 138.66, 150.78, 155.05, 155.52, 156.81, 157.11, 190.26. IR (KBr) ν_max_ 3429, 3356 (2NH), 1715, 1698, 1658 (3C=O) cm^−1^. MS *m/z* (%) 451 (M^+^ + 1, 1), 450 (M^+^, 5), 303 (21), 104 (100), 76 (41). Anal. Calcd for C_25_H_18_N_6_O_3_ (450.45): C, 66.66; H, 4.03; N, 18.66; Found: C, 66.48, H, 4.20, N, 18.50%.

*3-(4-Ethoxycarbonylphenylhydrazono)-4-phenyl-[1,2,4]triazepino[2,3-a]quinazoline-2,7(1H)-dione* (**6j**). This compound was obtained as reddish brown solid, (75%). mp. 180–182 °C ^1^H-NMR (DMSO-d_6_) δ 1.30 (t, *J* = 7 *Hz*, 3H, CH_3_ ), 4.26 (q, *J* = 7 *Hz*, 2H, CH_2_), 7.23 (d, *J* = 8 *Hz*, 2H, ArH), 7.43–7.99 (m, 9H, ArH), 8.26 (d, *J* = 8 *Hz*, 2H, ArH), 11.10 (s, 1H, NH), 12.12 (s, 1H, NH); IR (KBr) ν_max_ 3394, 3150 (2NH), 1725, 1681, 1643 (3C=O) cm^−1^. MS *m/z* (%) 481 (M^+^ + 1, 3), 480 (M^+^, 16), 479 (10), 105 (100), 104 (17), 77 (43). Anal. Calcd for C_26_H_20_N_6_O_4_ (480.47): C, 64.99; H, 4.20.; N, 17.49; Found: C, 64.84, H, 4.10, N, 17.35%.

### 3.2. Agar Diffusion Well Method to Determine the Antimicrobial Activity

The microorganism inoculums were uniformly spread using sterile cotton swab on a sterile Petri dish containing Malt extract agar (for fungi) and nutrient agar (for bacteria). Each sample (100 μL) was added to each well (6 mm diameter holes cut in the agar gel, 20 mm apart from one another). The systems were incubated for 24–48 h at 37 °C (for bacteria) and at 28 °C (for fungi). After incubation, microorganism growth was observed. Inhibition of the bacterial and fungal growth were measured in mm. Tests were performed in triplicate p [31].

## 4. Conclusions

In this report, a simple method for the synthesis of 3-arylhydrazono-4-phenyl-[1,2,4]triazepino[2,3-*a*]-quinazoline-2,7(1*H*)-dione was accomplished by the coupling of 4-phenyl-1,3-dihydro[1,2,4]-triazepino[2,3-a]quinazolin-2,7-dione and the diazonium salt of aniline derivatives. The tautomeric structure of the products was investigated. Their spectral data indicate collectively that such compounds exist predominantly in the hydrazone tautomeric form **6A**. In addition, the antimicrobial activity of some of the products showed moderate activities against *E. coli* and *S. aureus* although these activities are far less than those of the studied reference drugs (tetracycline and amphotericin B).
